# Molecular mechanisms of ferroptosis in ulcerative colitis: insights from machine learning, WGCNA, and immune cell infiltration analysis

**DOI:** 10.3389/fimmu.2025.1615186

**Published:** 2025-08-29

**Authors:** Leilei Zhai, Huiyue Pan, Ziyi Guo, Wei Zhou, Qi Ding, Haikun Wang, Qian Chen, Ping Yao

**Affiliations:** ^1^ The First Department of Gastroenterology, The First Affiliated Hospital of Xinjiang Medical University, Urumqi, Xinjiang Uygur Autonomous Region, China; ^2^ Department of Nephrology, The Children’s Hospital, Zhejiang University School of Medicine, National Clinical Research Center for Child Health, Hangzhou, China

**Keywords:** ulcerative colitis, machine learning, ferroptosis, WGCNA, immune infiltration, MFN2, CBS

## Abstract

**Background:**

This study aimed to investigate ferroptosis-related biomarkers and their potential molecular basis in UC.

**Methods:**

UC datasets (GSE87466 and GSE47908) from the Gene Expression Omnibus database were merged as the training set, and batch effects were removed. Ferroptosis-related differentially expressed genes (DE-FRGs) were selected to construct a diagnostic risk model in UC. Machine learning (lasso regression and SVM-RFE), Weighted Gene Co-expression Network Analysis (WGCNA) and PPI were then used to obtain candidate hub genes. After identifying common DE-FRGs, functional enrichment analysis, GSEA and GSVA functional enrichment analysis and immune cell infiltration were performed to explore the pathogenesis of UC. Besides, the correlation of hub gene expression and ferroptosis signature markers (GPX4 and ACSL4) was validated in external validation (GSE92415) and *in vitro* experiments. Finally, we employed the human intestinal epithelial Caco-2 cell to establish an *in vitro* inflammatory model by treatment with LPS (1 μg/ml) for 24 hours. This model was used to validate the correlation between the expression levels of ferroptosis-related essential genes (ACSL4 and GPX4) and pro-inflammatory cytokines (TNF-α, IL-6, and IL-1β). Furthermore, to confirm ferroptosis involvement, Caco-2 cells were co-treated with RSL3 (a ferroptosis inducer) or Ferrostatin-1 (Fer-1, an inhibitor), followed by measurement of GSH, MDA as an indicator of lipid peroxidation, and cellular iron load. Mitochondrial ultrastructure was assessed via transmission electron microscopy (TEM) to detect ferroptosis-associated morphological changes.

**Results:**

MFN2 and CBS were identified as hub genes after further validation. Functional estimation, gene set enrichment analysis, and immune infiltration signature identification showed notable associations of the hub genes with macrophages, mast cells resting, and follicular helper T cell levels. *In vitro*, we observed that treatment with LPS/RSL3 obviously activated ferroptosis in Caco-2 cells, as indicated by altered expression of key ferroptosis-related genes (down-regulation of GPX4, CBS, and MFN2; up-regulation of ACSL4) and the levels of surrogate ferroptosis markers (elevated MDA and iron levels, along with reduced GSH). In addition, LPS-induced ferroptosis in Caco-2 cells could be reversed by Fer-1.

**Conclusions:**

MFN2 and CBS may represent potential therapeutic targets and could serve as biomarkers for immune regulation in UC, warranting further investigation.

## Introduction

The etiology of ulcerative colitis (UC), a chronic inflammatory disorder primarily affecting the colon and characterized by recurrent cycles of inflammation and remission, remains unclear (1). Typically initiated in the rectum, the inflammation may extend proximally along the colon. A multitude of factors, including genetic predisposition, dysfunction of the intestinal barrier, dysregulated immune responses, and environmental triggers, are believed to contribute to the pathogenesis of UC (2). Notably, the rising prevalence of UC in developing countries poses a significant public health concern (3). Ferroptosis, a regulated form of cell death characterized by the iron-dependent accumulation of intracellular lipid reactive oxygen species (ROS) leading to membrane damage (4), is intricately linked to various biological processes, such as amino acid, iron, and polyunsaturated fatty acid (PUFA) metabolism (5). While the precise role of ferroptosis in UC pathogenesis remains unclear, emerging evidence suggests its involvement in intestinal barrier injury and immune activation (6). This immune activation further damages the intestinal barrier, creating a vicious cycle of inflammation and barrier dysfunction. Researchers have found that ferrostatin-1 (Fer-1), a specific ferroptosis inhibitor, can effectively ameliorate DSS-induced UC by negatively regulating the Nrf2/HO-1 signaling pathway (7).

Additionally, the interplay between ferroptosis and the immune system is significant in UC (8). The majority of the intestinal wall’s lamina propria macrophages during the active phase of UC have the M1 phenotype. M1 causes the epithelial barrier to be destroyed, tight junction proteins to be broken down, epithelial cells to undergo apoptosis, and inflammation to become excessive (9). The infiltration of macrophages and the release of pro-inflammatory factors promote the carcinogenesis of epithelial cells (10). Neutrophils play a primary function in the development and maintenance of intestinal inflammation. Chemokines and reactive oxygen species (ROS) are produced, the epithelial barrier is disrupted, other immune cells are recruited and activated, and redox-sensitive inflammatory pathways are activated (11). The death of epithelial cells due to ferroptosis can trigger an immune response, leading to the activation of immune cells and the release of pro-inflammatory cytokines. The gut microbiota also plays a role in this process, as dysbiosis can exacerbate ferroptosis and immune activation, contributing to the progression of UC (12, 13). Additionally, investigating immune infiltration in UC and how it relates to ferroptosis will aid in the analysis of the pathophysiology of ferroptosis in UC. Inhibiting ferroptosis may be a novel way to stop the disease from getting worse. Although its precise pathophysiological significance is yet unknown, ferroptosis has a substantial correlation with several disorders, including UC (14). Therefore, a better understanding of the molecular basis of ferroptosis is crucial for improving targeted ferroptosis-based treatment options. However, related biomarkers that regulate intestinal epithelial cell (IEC) ferroptosis in UC have not been fully elucidated.

The expression profile and relationship of ferroptosis-related genes (FRGs) in UC remain unknown. Therefore, this study intended to identify key FRGs and evaluate their value for UC diagnosis. We intersected these differentially expressed genes (DEGs) from the GEO database in UC with FRGs to find ferroptosis-related DEGs (DE-FRGs). A risk model was constructed using the DE-FRGs via machine learning methods, and a PPI network and Weighted Gene Co-expression Network Analysis (WGCNA) were constructed to assess the diagnostic hub genes. Moreover, the correlations between the key DE-FRGs with immune infiltration and relevant networks were explored.

Furthermore, the identified potential biomarkers will be systematically validated through external cohort analysis and *in vitro* functional experiments. To elucidate the role of ferroptosis in this process, cells will be subjected to treatment with either lipopolysaccharides (LPS), RSL3 (a ferroptosis inducer), or Ferrostatin-1 (Fer-1, a ferroptosis inhibitor), followed by a comprehensive assessment of key ferroptosis markers, including glutathione (GSH), malondialdehyde (MDA), and intracellular iron concentrations. Ultimately, this study aims to identify and characterize novel ferroptosis-related biomarkers, which may facilitate the development of targeted biotherapies for UC.

## Materials and methods

### GEO datasets downloading and preprocessing

**Figure 1:**
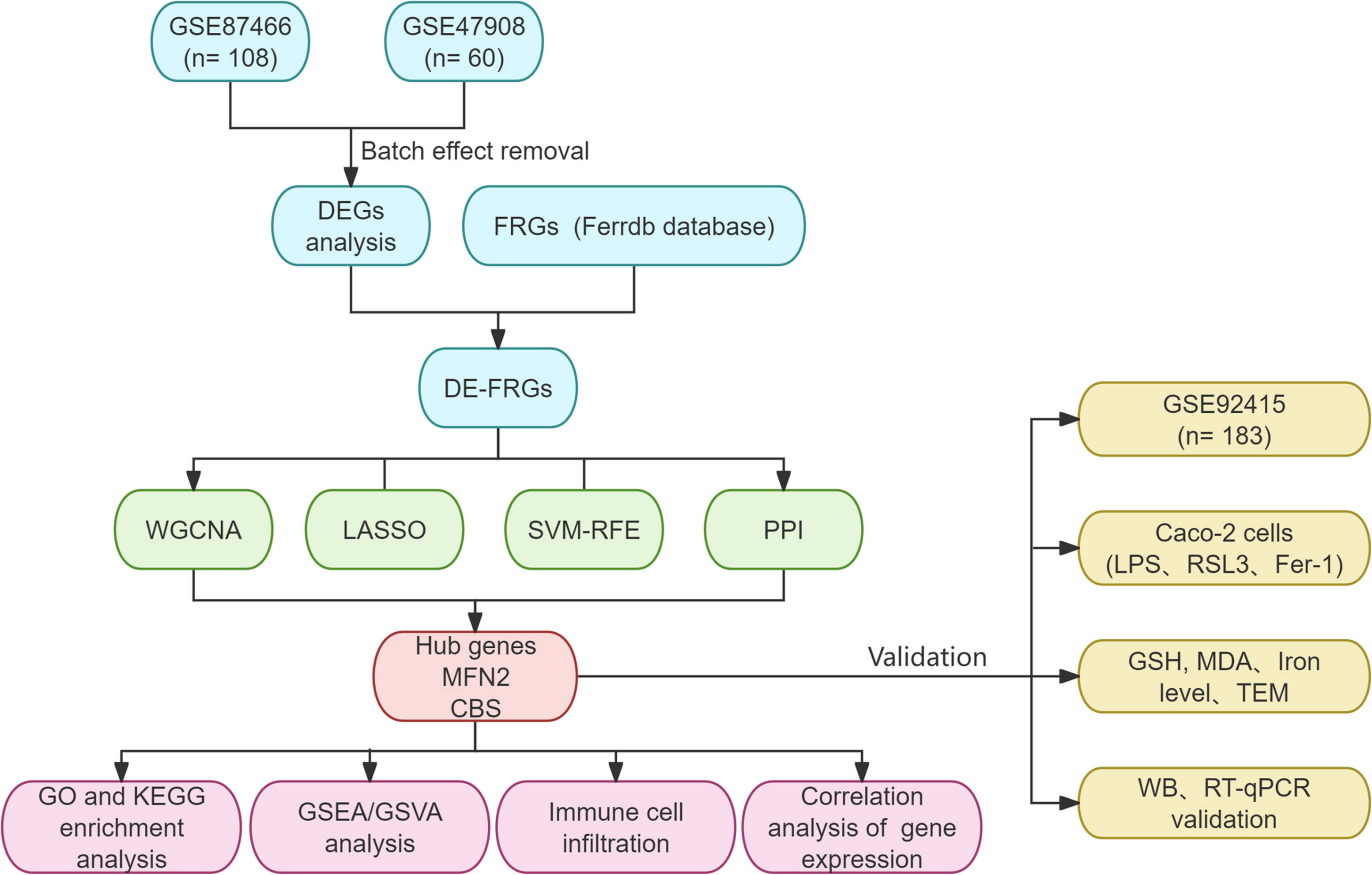
Demonstrates the flowchart of this study.

Datasets were downloaded from the GEO databases (https://www.ncbi.nlm.nih.gov/geo/). We performed a systematic search and filtered them according to the following criteria: 1) Homo sapiens; 2) expression profiling by array; 3) UC patients and normal controls were included in the samples; 4) the sample was from colonic mucosal tissue; 5) there was no statistically significant difference in baseline data between the groups. Subsequently, GSE87466 (87 UC, 21 controls) and GSE47908 (45 UC, 15 controls) were combined as a training set (**Table 1**). The comBat function of the “sva” R package was performed to eliminate the batch effect in the gene expression profiles (15, 16). We then obtained the UC-related data set and the expression matrix of the Principal Component Analysis (PCA). PCA is the preferred method to reduce the data dimension and analyze the effect of batch effect removal. GSE92415 was designated as the validation set. The flowchart is shown in **Figure 1**. Background calibration, data normalization, and log2 transformation were performed on the included datasets using “affy” in R software (version 4.1.2, https://www.r-project.org/).

**Table 1:** Microarray information.

GEO ID	Platform	UC	Controls	Source tissue	Attribute
GSE87466	GPL13158	87	21	colon	Training set
GSE47908	GPL570	45	15	colon	Training set
GSE92415	GPL13158	162	21	colon	Validation set

GEO, Gene Expression Omnibus; UC, ulcerative colitis.

### Ferroptosis-related DEGs identification

Differential expression analysis was carried out on the elected datasets using the R software’s “limma” package (17). The DEGs were then classified according to the criteria of adjusted *P* value <0.05 (18). The ggplot2 package was utilized to visualize the heatmaps and volcano maps of DEGs. Ferroptosis-related genes (FRGs) were searched in the Ferrdb database (http://www.zhounan.org/ferrdb/) (19). The database contains four types of ferroptosis-related genes: Driver, Suppressor, Marker, and Unclassified. Genes associated with ferroptosis were retrieved from each category and duplicates were removed before creating a consolidated list.

### Function enrichment analysis

To evaluate the interactions between proteins and genes, pathways, co-expression, co-localization, and protein domain similarities, GeneMANIA (http://genemania.org) analyses were conducted (20). GO is a database set up by the Gene Ontology Consortium, which aims to define and describe the functions of genes and proteins for a variety of species. GO functional enrichment analysis and KEGG pathway enrichment analysis were carried out to elucidate the functions and the enriched pathways of the potential DEGs.

### Weighted gene co-expression network analysis

To identify potential disease biomarkers or therapeutic targets, WGCNA is used to discover modules of highly correlated genes and describe the relationships between modules and correlations with external sample features (21). The differential gene expression data of merged UC-related are analyzed using WGCNA and R software (version 4.3.1). First, the optimal soft threshold was established, and an unscaled network was built. This network was converted to a weighted network at a soft threshold power of 5, and modules were found using hierarchical clustering (minModuleSize = 30, mergeCutHeight = 0.25). Subsequently, the adjacency relationship was converted into a topological overlap matrix (TOM), which demonstrated the shared neighboring genes’ concurrence. It was also determined what the relevant dissimilarity degree (1-TOM) was. Ultimately, dynamic tree-cut algorithms and hierarchical clustering were used to discover modules. A network cluster dendrogram was used to illustrate the clustering. To ascertain the relationship with UC, typical gene expression levels were computed and displayed from co-expressed merging modules indicated by color. In order to identify important linkages in UC, the analysis’s main goal is to compute the correlation between these modules and sample groups.

### Identification of hub genes by lasso regression and SVM-RFE

In this study, the least absolute shrinkage and selection operator (LASSO) in conjunction with support vector machine-recursive feature elimination (SVM-RFE) machine learning algorithm was applied to screen for UC-related characteristic genes. With the “glmnet” package in R (15), LASSO is a regularized regression technique. A method of supervised machine learning called SVM-RFE may prioritize features according to recursion in order to prevent overfitting (16, 17). The genes that show up as overlaps between the genes examined by the two machine learning techniques are potential bio-diagnostic indicators. Protein-protein Interaction (PPI) network analysis. The PPI network was derived based on the STRING database (https://string-db.org) (22). The selecting interaction threshold was “highest confidence (0.4)”. Visualization of these results was performed with “Cytoscape” (version 3.10.0). Subsequently, we identified densely connected network components using the cytoHubba plugin and degree algorithm.

### Screening hub genes and external validation

Ultimately, a Venn diagram was drawn to identify as diagnostic biomarkers the intersection between the DE-FRGs obtained from the LASSO, SVM-RFE, PPI, and WGCNA. The external testing cohorts provided additional confirmation of the biomarkers’ differential expression and predicted reliability (GSE92415). Receiver operating characteristic (ROC) curves were drawn by pROC, and the predictive usefulness of the discovered biomarkers was estimated by computing the area under the ROC curve (AUC) value. Using a box plot, we demonstrate the hub DE-FRG expression between the UC populations and controls. Scatterplots were made to show the correlation between the hub genes and ferroptosis markers such as ACSL4 and GPX4.

### GSEA, ssGSVA function enrichment analysis and immune infiltration enrichment analyses

GSEA is a computational method used to assess whether there is a statistically significant and consistent difference between two biological data sets in a preset gene set. We used the clusterProfiler package to analyze GSEA gene functional enrichment in hub genes (grouped by median expression level). The single-sample GSEA (ssGSEA) algorithm and single-sample gene set variation analysis (ssGSVA) were utilized to evaluate signaling pathways associated with signature genes (23). The relative content and dynamic regulatory process of each of the 22 immune cell types was determined using the CIBERSORT method (24). Immunoinfiltration analysis was performed using the CIBERSORT algorithm with 1,000 permutations on the variations in immune cell expression between UC and healthy controls. Statistical significance was assessed by permutation test, using *P* < 0.05 to screen for significant infiltration between subsets. To visualize the correlation between the enrichment levels of 22 infiltrating immune cells and the expressions of the diagnostic genes, use the “linkET” package to create correlation heat maps. To view the differences between the immune cells in the UC group and the controls, use the “boxplot” package to create a violin chart. Spearman’s correlation analysis was conducted to explore the association between the diagnostic biomarkers and immune cell infiltration in the colon tissue.

### Cell culture and cell viability assay

The STR analysis was conducted to verify the authenticity of human epithelial cells line Caco-2 obtained from Procell (Procell Life Science & Technology Co., Ltd., China). During the culture, cells were maintained in MEM supplemented at 37°C in a humidified environment with 5% CO_2_, 20% FBS, penicillin (100 U/mL), and streptomycin (100 mg/mL). Caco-2 cells were seeded into 96-well microplates at a density of 1×10^5 cells/mL. Using the CCK8 assay, the viability of lipopolysaccharides-treated (LPS) doses of 0.1, 0.25, 0.5, 1, 2.5, 5, and 10 µg/mL was detected to determine the appropriate concentration to use in subsequent experiments. After a 24-hour exposure to LPS, cells underwent a 2-hour incubation with CCK8 at a 10% concentration. At 450 nm, the absorbance (OD) was calculated utilizing a microplate reader (Thermo Scientific, Waltham, United States). In order to observe the effect of LPS on ferroptosis, Caco-2 cells were separated into 4 groups: control group, model group (LPS, 1 μg/mL) (25), RSL3 group (15μM) (26), and LPS+Fer-1 group (4 μM) (27), and Malondialdehyde (MDA), glutathione (GSH), and iron load levels were measured. LPS derived from Escherichia coli O55:B5 (HY-D1056), Ferrostatin-1 (Fer-1, HY-100579), and RSL3 (HY-100218A) were obtained from MedChemExpress (Monmouth Junction, NJ, USA).

### Measurement of MDA, GSH, and Fe^2+^ levels

Caco-2 cells were lysed with the corresponding lysis buffer, and the supernatant was used in the assay kit after sonication and centrifugation. MDA content was measured using the MDA Content Assay Kit (BC6415, Solarbio, China). Fe^2+^ levels were quantified with the Ferrous Ion Content Assay Kit (BC5415, Solarbio, China). GSH concentration was assessed using the Reduced Glutathione (GSH) Assay Kit (A006-2-1, Jiancheng Bioengineering Institute, Nanjing, China).

### Transmission electron microscope

Caco-2 cells were trypsinized, washed twice with PBS, and fixed for 30 minutes in ice-cold glutaraldehyde. After being washed with PBS, the cells underwent post-fixation in 1% osmium tetroxide and were dehydrated in graded ethanol and Epon embedded. Cut sections measuring 60–80 nm in thickness with an ultramicrotome. Thin sections were examined under a transmission electron microscope (H7700, Transmission Electron Microscope; Hitachi, Japan).

### The RNA extraction and real-time quantitative PCR

Total RNA was extracted using Foregene RNA isolation kit (Foregene Co.Ltd, China). RNA extracts were reverse-transcribed into cDNA with PrimeScript RT Reagent Kit (Takara, Shiga, Japan). RT-qPCR was performed by using SYBR Green PCR Master Mix (TransGen Biotech, China), and the detection of qPCR was performed on an ABI QuantStudio5 using GAPDH as an internal control. All primers were crafted and manufactured by Sangon Biotech, located in Shanghai, China, and **Supplementary Table 1** contains their sequences. The 2^-ΔΔCT^ method was used to analyze the relative data, while statistical analysis was conducted using T-tests.

### Western blotting

After lysing the Caco-2 cells in ice-cold RIPA buffer (PC101, Epizyme, Shanghai, China). Using a combination of protease and phosphorylase inhibitors (Epizyme, Shanghai, GRF103), total protein was extracted from cells using RIPA buffer, and the BCA protein assay kit (ZJ102L, Epizyme) was used to quantify the protein. Subsequently, the total protein was then separated using sodium dodecyl sulfate-polyacrylamide gel electrophoresis (SDS-PAGE). Preparation of the gel involved a 10% polyacrylamide gel electrophoresis kit (PG112, EpiZyme). Following their separation, the proteins were put onto PVDF (polyvinylidene fluoride) membranes (IPVH00010, Millipore, USA) in order to be further immunoblotted using certain antibodies. Primary antibodies were as follows: MFN2 (1:1000, CST #9482), CBS (1:1000, CST #14782), GAPDH (ABclonal #A19056, 1:50000), GPX4 (ABclonal #A11243, 1:1000) and ACSL4 (ABclonal #A20414, 1:1000). Incubation with primary antibodies overnight was followed by incubation with HRP-conjugated secondary antibodies at room temperature for an hour (ABclonal #AS014). The eBlot Touch Imager (eBlot, Shanghai, China) was employed to detect protein bands using Omni-ECL enhanced chemiluminescence reagent kit (SQ101, Epizyme, Shanghai, China). Gray values were analyzed using ImageJ software, and protein relative expression was calculated by comparing the certain protein gray values to internal reference proteins.

### Statistical analysis

Data analysis and visualization were performed using GraphPad Prism software (Version 8.0, San Diego, CA) and R software (version 4.1.2). The results were expressed as the mean ± SD of three independent experiments. The comparison between the two groups was performed by an unpaired, two-tailed Student’s t-test or chi-square tests. Two-way analysis of variance (ANOVA) with a *post-hoc* test was applied for multigroup comparisons. Asterisk * denoted the statistical significance threshold of p < 0.05, ** denoted p < 0.01, and ^#^ denoted p < 0.001.

## Results

### Screening of DE-FRGs in UC

**Figure 2:**
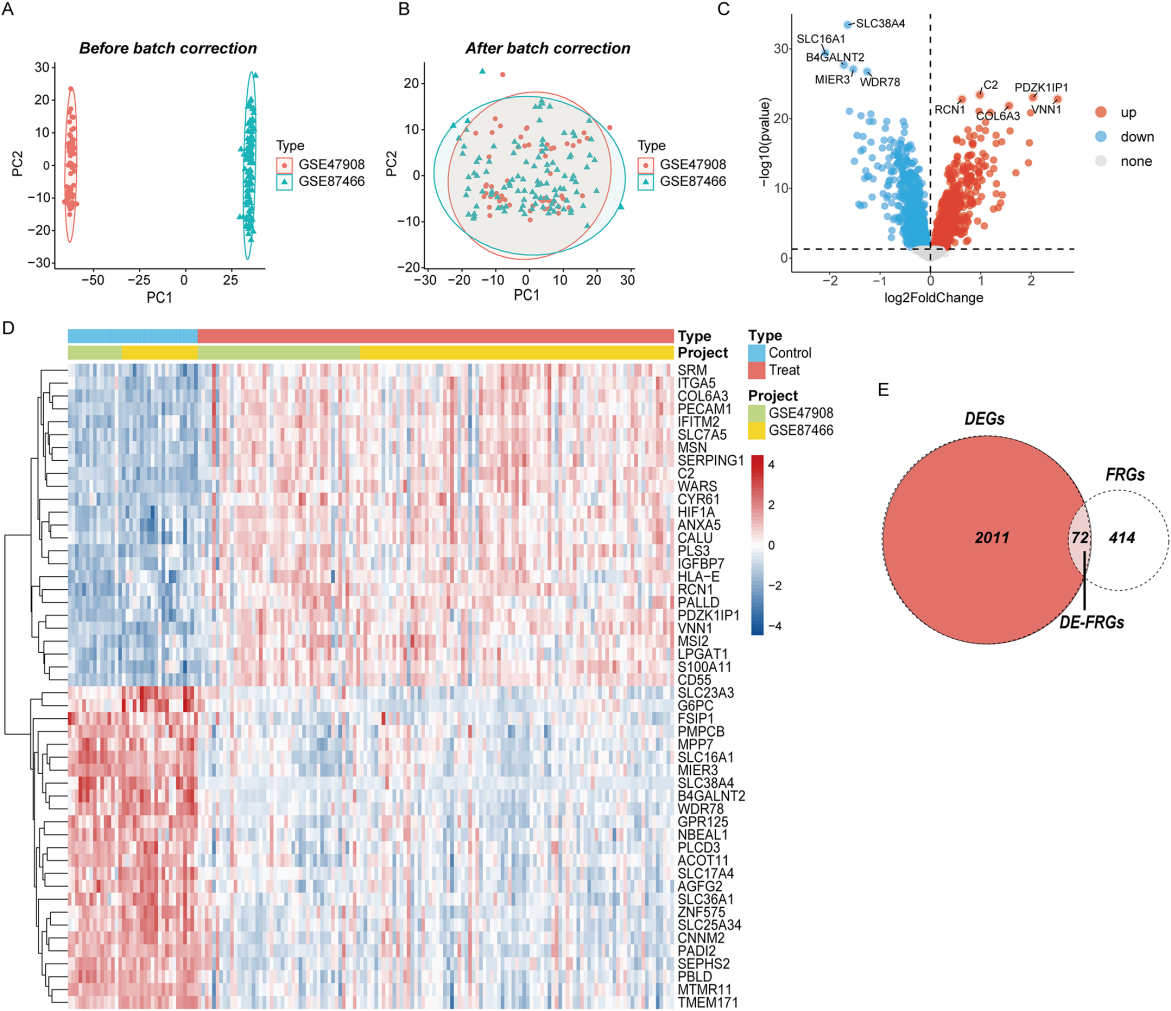
Eliminating the batch effect between different sequencing platforms. **(A)** The PCA plot before elimination of batch effect, **(B)** is the PCA plot with batch effect elimination; **(C)** the volcano map of DEGs in UC, and **(D)** the top 50 up-regulated and down-regulated genes of UC, **(D)** the veen plot of DE-FRGs.

The gene expression matrices GSE87466 and GSE47908 were acquired from the GEO database and merged as the training set, which available for gene level data of 132 UC patients and 36 control samples. PCA showed clear differences between these two clusters (**Figure 2A**). After correction, as displayed in **Figure 2B**, all samples in the dataset achieved acceptable homogeneity following PCA analysis. The distribution and variations of all gene expression between the UC and control groups are displayed in boxplots (**Supplementary Figure 1**). DEGs were visualized using a volcano map (**Figure 2C**) showing the top 50 DEGs in the heatmap (**Figure 2D**). By intersecting DEGs and FRGs, we identified 72 DE-FRGs linked to UC (**Figure 2E**).

### Weighted correlation network analysis

**Figure 3:**
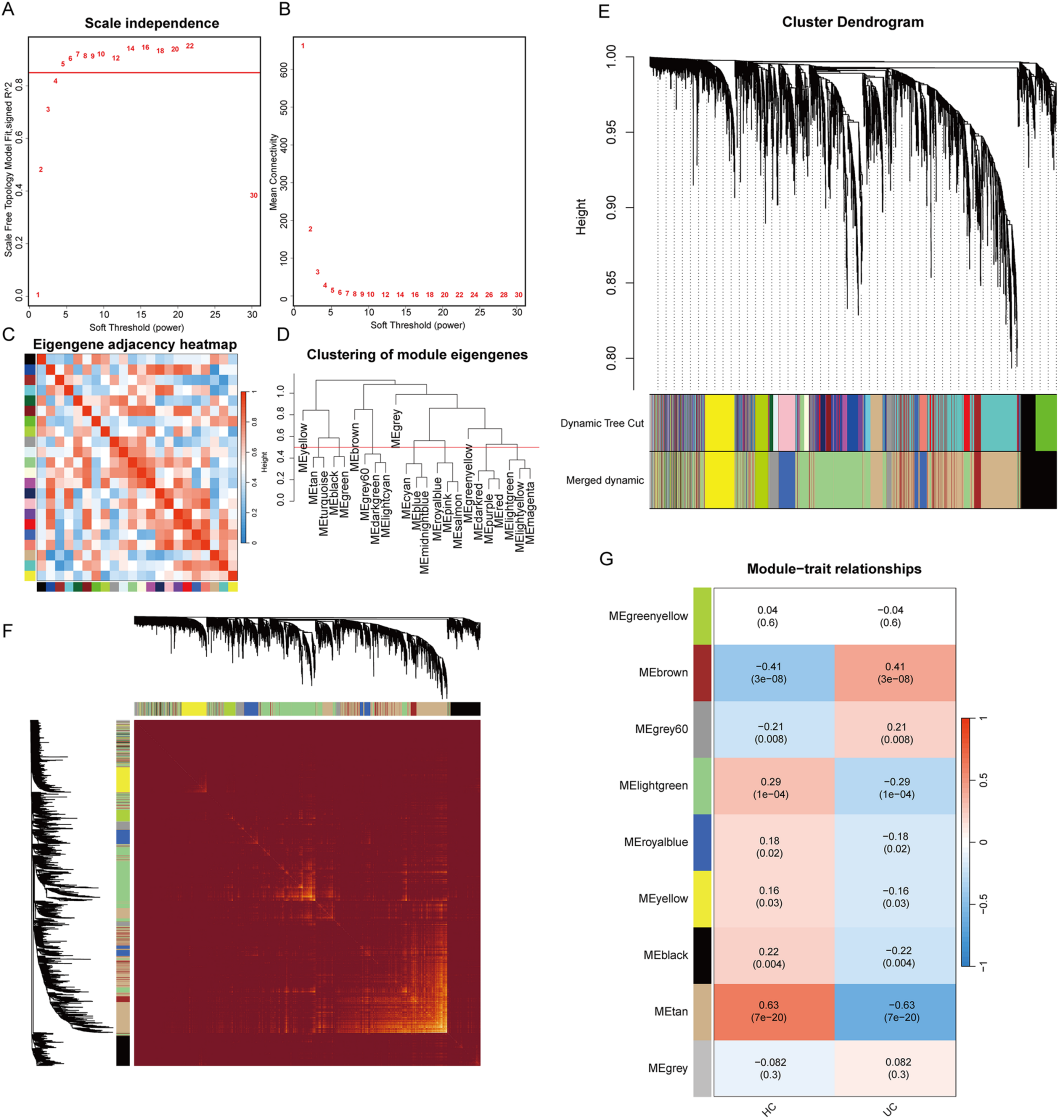
Results of the WGCNA analysis. **(A)** analysis of the scale-free fit index for various soft threshold powers (β); **(B)** analysis of the mean connectivity for various soft threshold powers; **(C)** heatmap of the correlation of modules; **(D)** cluster dendrogram of modules; **(E)** cluster dendrogram of genes; **(F)** adjacency heatmap of eigengenes; **(G)** correlations between different modules and clinical traits.

After removing the aberrant samples and filtering genes, WGCNA was constructed, containing 4111 genes and 168 samples. When the scale-free fit index was 0.85, the minimum soft-thresholding power was 5 (**Figures 3A, B**), indicative of an approximate scale-free topology. The samples and each module were analyzed using heat maps (**Figures 3C, D**). In total, dynamic tree cutting yielded nine distinct co-expression modules that were merged and identified by a unique color (**Figure 3E**). The eigengene adjacency heatmap is shown in **Figure 3F**. Subsequently, the association between each module and the clinical features was examined, which indicates that the MEtan module (1,175 genes) was the most associated with UC (r = 0.63; P = 7e-20), whereas it was selected for additional research (**Figure 3G**). Sample clustering and more details are provided in **Supplementary Figure 2**.

### Screening for diagnostic biological markers

**Figure 4:**
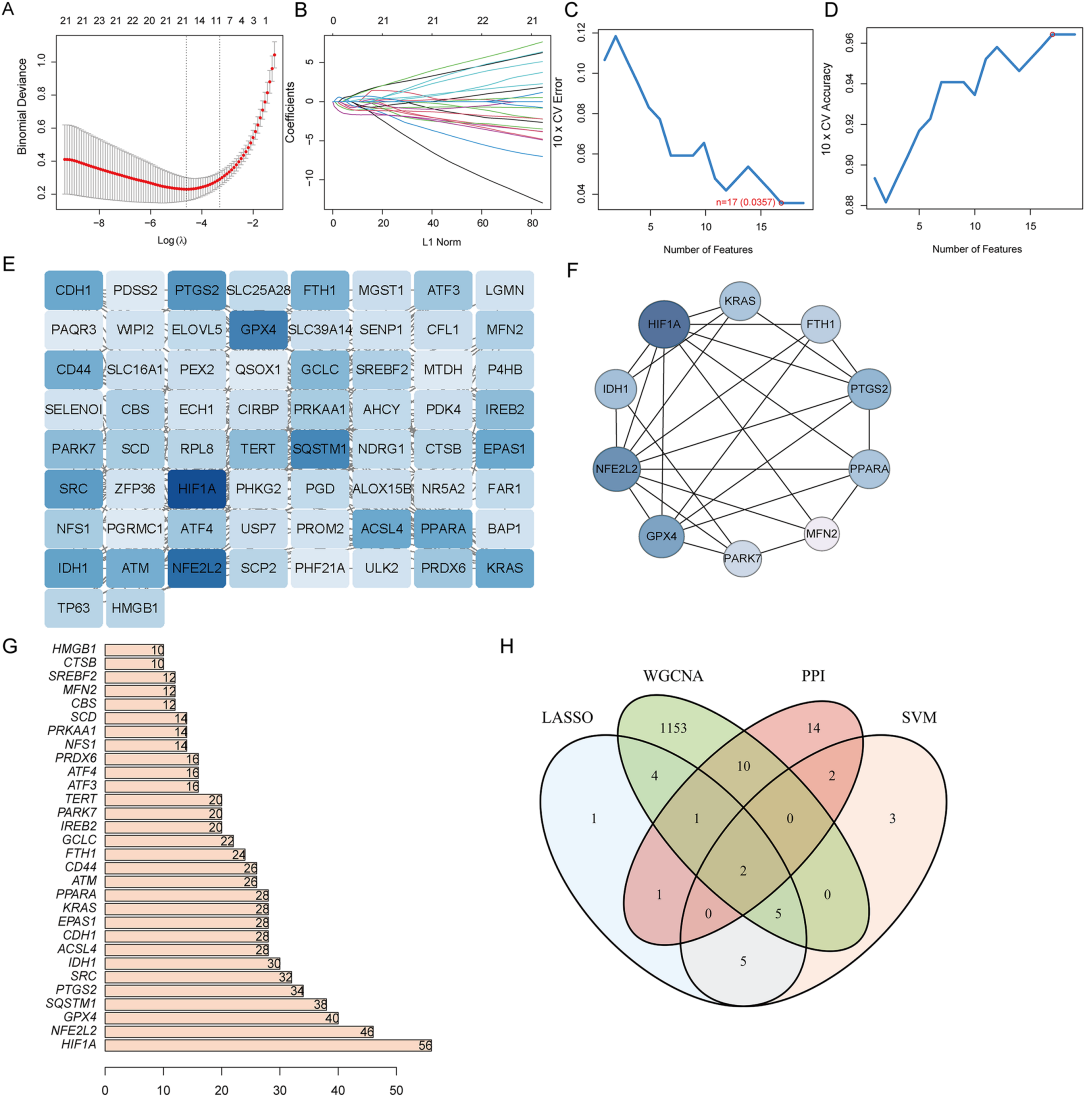
Narrowing down candidate biomarkers by LASSO analysis and SVM-REF. **(A, B)** LASSO analysis. **(C, D)** SVM-REF analysis to identify signature genes; **(E)** PPI network module results; **(F)** PPI network established by using CytoHubba; **(G)** PPI network constructed by using Degree algorithm; **(H)** Venn diagram of intersecting genes by LASSO, SVM-REF, WGCNA, and PPI network.

Two machine learning algorithms were employed to identify hub genes in DE-FRGS, incorporating lasso regression and SVM. Utilizing the LASSO algorithm in conjunction with 10-fold cross-validation, the number of genes corresponding to the minimum cross-validation error was determined (**Figures 4A, B**). The optimal number of genes corresponding to the minimum cross-validation error was selected by employing the SVM-RFE algorithm and conducting 10-fold cross-validation (**Figures 4C, D**) (**Supplementary Table 4**). The PPI network was then utilized to identify hub genes including 66 nodes and 229 interactional pairs (**Figure 4E**). The identification of hub genes was subsequently carried out using the CytoHubba plugin (**Figure 4F**). To visualize and screen the top 30 hub genes, the constructed PPI network was imported into Cytoscape and analyzed with the Degree algorithm (**Figure 4G**). WGCNA was then used to screen the Mtan cluster in UC. Finally, the intersection of the four aforementioned algorithms yielded the optimal gene signature, consisting of two diagnostic genes, MFN2 and CBS (**Figure 4H**).

### GO and KEGG function enrichment analysis of DE-FRGs

**Figure 5:**
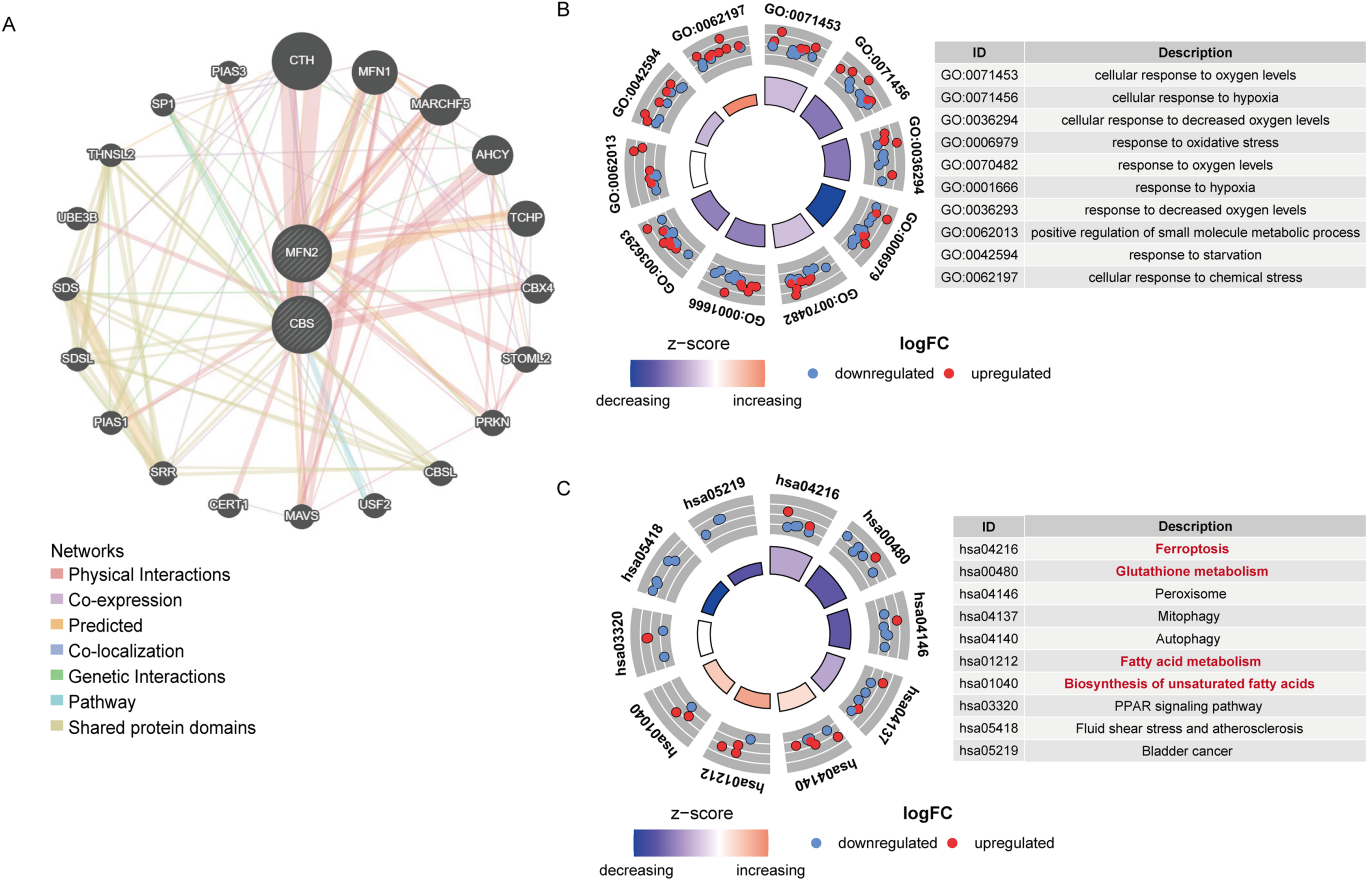
**(A)** The gene-gene interaction network of hub genes was analyzed by GeneMANIA. **(B)** Circos diagram of enriched GO terms. **(C)** Circos diagram of the KEGG pathways enrichment analysis.

Finally, we explored two hub genes (CBS and MFN2) and their 20 interacting genes using the GeneMANIA database (**Figure 5A**). The gene-gene interaction network for hub genes was analyzed using the GeneMANIA database.

GO and KEGG enrichment analyses of the DE-FRGs were conducted. The results are illustrated in **Figures 5B, C**. Response to oxidative stress (GO:0006979) was tightly related to UC biological processes (**Figure 5B**). The main disease-related terms in cellular components consisted of peroxisome (GO:0005777), microbody (GO:0042579), and peroxisomal matrix (GO:0005782). Concerning molecular functions, more significant enrichment was found in peroxidase activity (GO:0004601). Meanwhile, as suggested by KEGG enrichment, the top 10 pathways were visualized in the bubble chart, such as the glutathione metabolism, ferroptosis, biosynthesis of unsaturated fatty acids, and fatty acid metabolism pathway (**Figure 5C**) More enrichment analysis results are in **Supplementary Tables 2**, **3**.

### GSEA analysis and immune cell infiltration

**Figure 6:**
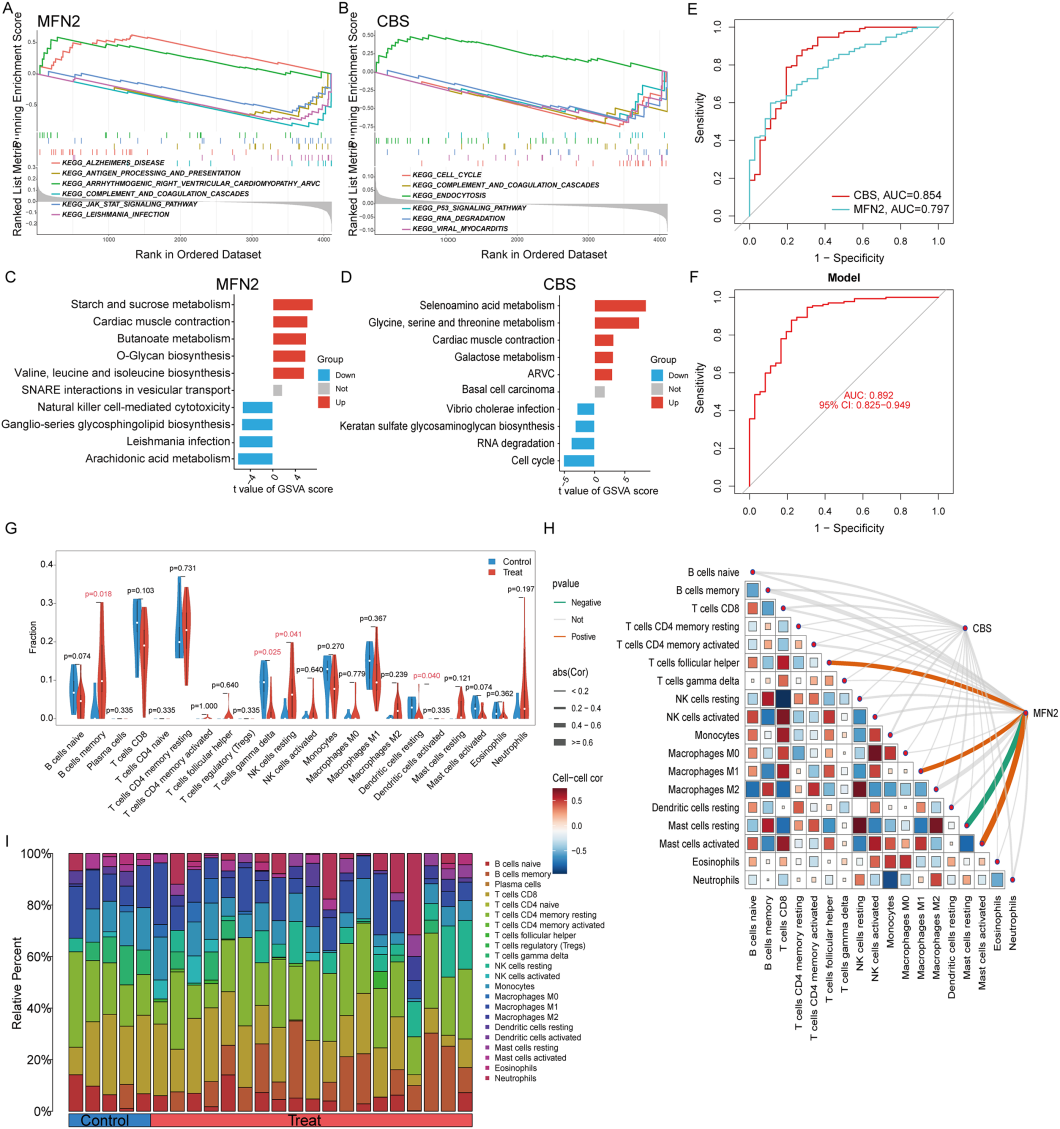
GSEA and GSVA enrichment analysis of hub genes: **(A)** GSEA results for MFN2; **(B)** GSEA results for CBS, **(C)** GSVA analysis of MFN2, **(D)** GSVA analysis of CBS. **(E)** ROC curve analysis of single differentially feature genes. **(F)** ROC curve analysis of the combined differential trait genes. **(G)** Split violin presented the different immune infiltration of 22 immune cells: lower T cells gamma delta (*P* = 0.025), resting dendritic cells (*P* = 0.040) and more B cells memory (*P* = 0.018) and NK cells resting (*P* = 0.041). **(H)** The association between signature genes and significantly different immune cell infiltration. **(H)** Displayed in a bar chart are the relative percentages of 22 immune cell subsets.

We assessed signaling pathways associated with signature genes via GSEA analysis. The top signaling pathways are displayed in **Figure 6**. The expression of CBS significantly correlated with cell cycle, complement and coagulation cascades, endocytosis, antigen processing and presentation, pathogenic *Escherichia coli* infection, and p53 signaling pathway. While MFN2 was significantly correlated with complement and coagulation cascades, antigen processing and presentation, the JAK-STAT signaling pathway, cytokine receptor interaction, natural killer cell-mediated cytotoxicity, cell adhesion molecules (CAMs), toll-like receptor signaling pathway (**Figures 6A, B**).

The ssGSVA analysis was conducted to investigate the characteristics of each feature gene regulatory pathway between UC and normal samples. We discovered after a thorough investigation that these genes were abundant in the cell cycle, immunological response, protein synthesis, and metabolic pathways, all of which were linked to ferroptosis in UC. The findings demonstrated that the overexpression of MFN2 transcription in the butanoate metabolism, O-glycan biosynthesis valine, leucine and isoleucine biosynthesis pathway, and the decreased level of it might induce the ferroptosis of UC by activating the arachidonic acid metabolism and natural killer cell-mediated cytotoxicity pathway (**Figure 6C**). While the high expression of CBS activated selenoamino acid metabolism glycine, serine and threonine metabolism, galactose metabolism pathway, and inhibited the keratan sulfate glycosaminoglycan biosynthesis, RNA degradation and cell cycle pathways (**Figure 6D**).

The signature genes were verified, the ROC curve was drawn, and AUC was compared to judge its diagnostic value. The AUC values of CBS and MFN2 were 0.854 and 0.797, which had certain diagnostic values. At the same time, through the comprehensive analysis of feature genes, the logical regression model was obtained, and the ROC curve was also drawn. We discovered that the complete model’s AUC value was 0.892, which was significantly higher than that of a single gene and suggested that it was more effective at predicting illnesses (**Figures 6E, F**).

Immunological features were evaluated according to immune cell infiltration. The relative content and dynamic regulatory process of each of the 22 immune cell types was determined using the CIBERSORT method (23). Analysis was also done on the variations in immune cell expression between UC and healthy controls. Compared with the healthy control group, immune cells had differential infiltration in UC patients, including lower T cells gamma delta (*P* = 0.025) and resting dendritic cells (*P* = 0.040) and more B cells memory (*P* = 0.018) and NK cells resting (*P* = 0.041) (**Figure 6G**). MFN2 was positively correlated with Macrophage M1, T cells follicular helper and Mast cells activated, while was negatively correlated with Mast cells resting (**Figure 6H**). Relative Percent of different immune infiltration among 22 immune cells showing in **Figure 6I**.

### Validation of hub gene expression

**Figure 7:**
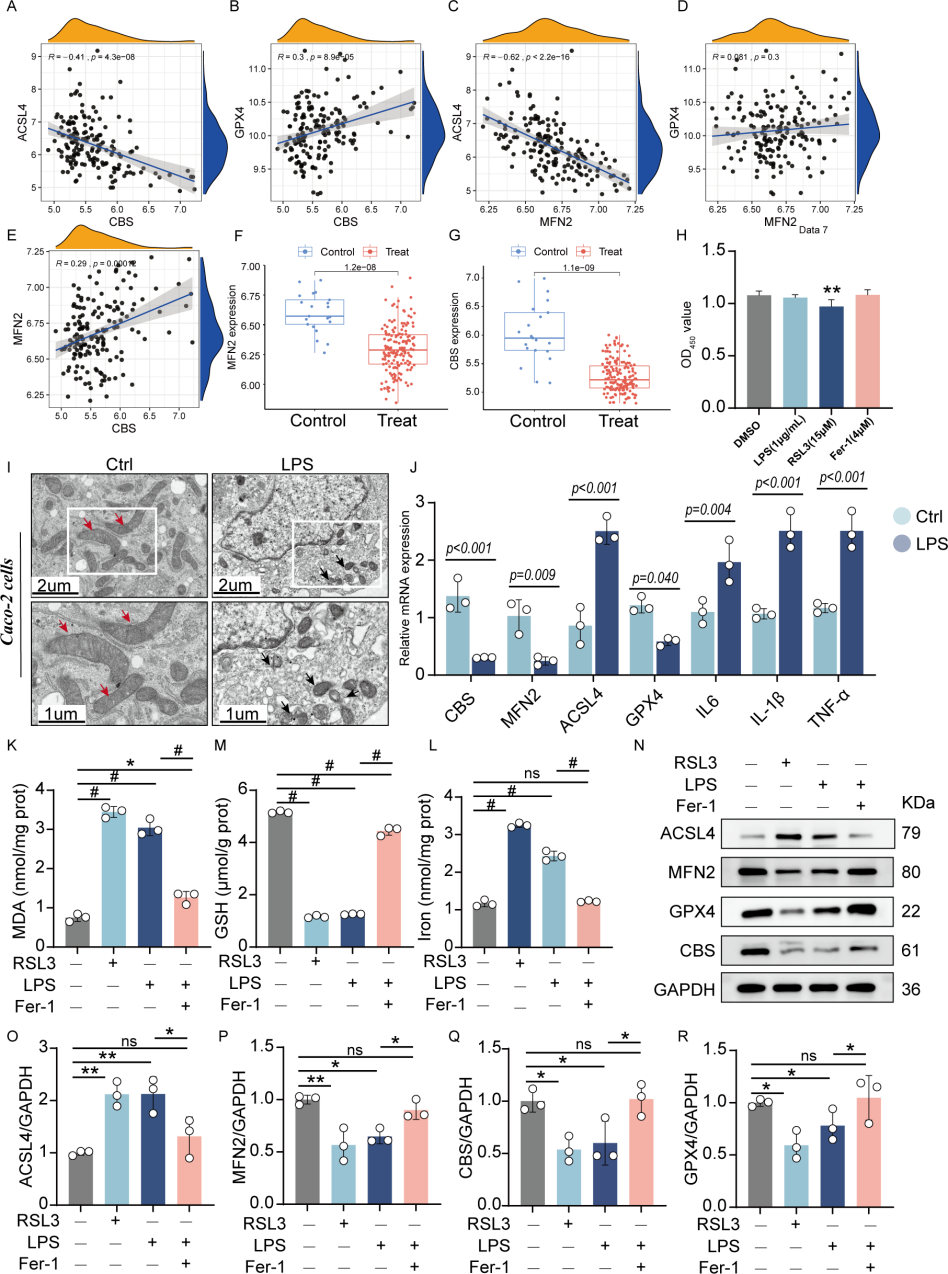
Validation of the hub genes: **(A-E)** Scatterplots of correlation between hub genes and characteristic ferroptosis markers (ACSL4 and GPX4). **(F, G)**. The expression level of hub genes in the validation sets (GSE92415). Cell viability of Caco-2 cells incubated with LPS (1 μg/mL), RSL3 (15μM), and Fer-1(4 μM) for 24h. **(I)** Representative TEM images of mitochondria: Red arrows indicate mitochondria with intact cristae, while black arrows highlight pathological mitochondrial alterations including vacuolization and cristae swelling. **(J)** The quantitative mRNA expression levels of TNF-α, IL-6, IL-1β, MFN2, CBS, ACSL4 andGPX4 in induced enteritis cells. The levels of MDA **(K)**, GSH **(M)**, and total iron **(L)** were quantified in Caco-2 cells following treatment with LPS, RSL3, or LPS+Fer-1. **(N)**. Representative protein expression bands: quantified expression levels of **(O)** ACSL4 **(P)** MFN2 **(Q)** CBS **(R)** GPX4 (n = 3, *P < 0.05, **P < 0.01, ^#^P < 0.001, ns, no significance, significantly different as indicated).

We chose the UC dataset GSE92415 as an external validation set to verify the expression of the candidate hub genes. Spearman’s correlation analysis was conducted to explore the association between the diagnostic biomarkers and immune cell infiltration in the colon tissue. Scatterplots show a significant, moderate correlation between the hub genes and ferroptosis markers such as ACSL4 and GPX4 (**Figures 7A-E**). Consistent with the results of the training set, MFN2 and CBS were significantly downregulated in the UC group (**Figures 7F, G**).

The LPS-stimulated Caco-2 cell line from human intestinal epithelial cells served as an *in vitro* model for the intestinal epithelium to check the association between the essential gene expression levels. As shown in the cytotoxicity assay, the viability of Caco-2 cells was significantly inhibited at RSL3 group (15μM), neither LPS group (1 μg/mL) nor Fer-1 group (4 μM) produced statistically significant changes in viability compared to controls (**Figure 7H**). For the obvious inhibition of cell viability at LPS doses of 2.5, 5, and 10 μg/mL after 24-hour exposure (0, 0.1, 0.25, 0.5, 1, 2.5, 5, and 10 μg/mL). In contrast, 1 μg/mL LPS did not exhibit significant cytotoxicity while still ensuring effective inflammatory stimulation (**Supplementary Figure 3**). Therefore, a subsequent experimental condition of 1 μg/mL LPS incubation for 24 hours was selected.

TEM revealed distinct ferroptotic morphology in LPS groups compared to controls, notably featuring shrunken mitochondria with disrupted cristae (**Figure 7I**). After 24 hours of induction of LPS, enteritis cells could secrete higher inflammatory factors TNF-α, IL-6 and IL-1β. RT-qPCR demonstrated that LPS exposure markedly decreased MFN2 and CBS mRNA expression but increased ACSL4 levels (**Figure 7J**).

Compared to control cells, LPS-treated Caco-2 cells exhibited significantly elevated iron levels and MDA accumulation, along with reduced GSH content - changes consistent with those observed in RSL3-induced ferroptosis (positive control). However, co-treatment with Fer-1 effectively attenuated these LPS-induced effects, as evidenced by decreased MDA content, reduced iron accumulation, and restored GSH levels. These findings demonstrate that LPS triggers ferroptosis in Caco-2 cells, which can be rescued by Fer-1 treatment (**Figures 7K, L**). Gene expression alterations were in agreement with the WB protein analysis results (**Figures 7N-R**). LPS treatment significantly upregulated ACSL4 expression while downregulating GPX4, MFN2, and CBS levels in Caco-2 cells. Notably, Fer-1 treatment effectively reversed these alterations by suppressing the LPS-induced ACSL4 overexpression and restoring GPX4, MFN2, and CBS expression. These results demonstrate that LPS-mediated downregulation of MFN2 and CBS activates the ferroptosis pathway and exacerbates inflammatory responses in intestinal epithelial cells. Fer-1 exerts its anti-ferroptotic effects at least partially through modulation of MFN2 and CBS signaling pathways.

In addition, it was determined that up-regulated ACSL4 expression, and had significant down-regulation effect on GPX4. These results suggest that down-regulation of MFN2 and CBS can activate the ferroptosis pathway and aggravate the inflammatory response of intestinal epithelial cells.

## Discussion

Ulcerative colitis is an intestinal inflammatory condition caused by genetic predisposition and environmental factors and is becoming more prevalent. It is known for its lengthy clinical progression and recurrent relapses (28, 29). Finding accurate molecular diagnostic biomarkers remains a challenge for UC. In this study, we performed an in-depth analysis of three GEO-derived datasets (GSE87466 and GSE47908 as training set and GSE92415 as validation set) to identify DEGs in UC patients. DE-FRGs were selected to construct a diagnostic risk model in UC. Next, machine learning (lasso regression and SVM-RFE), WGCNA, and PPI were screened to obtain candidate hub genes. Comprehensive functional analyses (GeneMANIA, GO, KEGG pathway enrichment, GSVA, GSEA function enrichment analysis) revealed several key biological processes and pathways associated with hub genes, including glutathione metabolism, ferroptosis, as well as the biosynthesis of unsaturated fatty acids and fatty acid metabolism pathways. CIBERSORT analysis highlighted a significant elevation in T cells gamma delta, dendritic cells resting, B cells memory, and NK cells resting within the colon tissues of UC patients. Finally, the expression of hub genes and ferroptosis feature changes were verified in external validation and *in vitro*.

To recap, we utilized a variety of bioinformatics tools to identify the two hub genes in UC, and their expression was confirmed in Caco-2 cells treated with LPS for 24 hours. These findings have the potential to help us better understand and explore the mechanisms behind the pathogenesis of UC. This could also aid in exploring potential biomarkers for identifying and treating patients with UC. Mitofusin 2 (MFN2) is a widely expressed mitochondrial transmembrane GTPase critical for mitochondrial fusion, and also contributes to maintaining the interorganelle contact sites between the endoplasmic reticulum and mitochondria. Mitochondrial fusion is mainly regulated by mitofusin 1 (MFN1), MFN2, and optic atrophy 1 (OPA1), whereas dynamin-related protein 1 (Drp1) and fission 1 (Fis 1) play an important role in mitochondrial fission (30). At present, emerging evidence has linked mitochondria to ferroptosis. Mitochondria are the major organelles for ROS generation and are responsible for iron metabolism and homeostasis (31). Simultaneously, mitochondria display decreased membrane potential and reactive oxygen species (ROS) accumulation due to damage to mitochondrial function (32). Impaired mitochondrial function leads to extensive production of ROS and free iron, promoting lipid peroxidation (33). Besides, mitochondria play a crucial role as an integrative platform for signal transduction, deciding whether cells undergo programmed cell death or continue to survive (34). Recent studies highlight the essential role of mitochondrial processes in the initiation and execution of ferroptosis, commonly referred to as mitochondria-dependent ferroptosis (35). Emerging evidence demonstrates that MFN2 serves as a multifunctional regulator capable of activating diverse signaling pathways. Notably, mitochondrial-targeted HO-1 has been shown to promote autophagy by facilitating Drp1 translocation to mitochondria. Furthermore, activation of the PKC-α/HO-1 pathway has been found to upregulate mitochondrial fusion proteins (Mfn1, Mfn2, and OPA1) while downregulating fission factors (Drp1 and Fis1) (36).

Cystathionine β-synthase (CBS) is a crucial enzyme in the transsulfuration pathway, which facilitates the conversion of homocysteine and serine into cystathionine. This cystathionine is subsequently transformed into cysteine by the enzyme cystathionine γ-lyase (CTH) (37). Cell proliferation and glutathione (GSH) synthesis are both dependent on cysteine (38), which is a non-essential amino acid. The reverse transsulfuration pathway is utilized by mammalian cells to synthesize cysteine in addition to acquiring exogenous cystine through system Xc^−^ (39, 40). The cysteine-limited tumor microenvironment necessitates cancer initiation and progression, and tumor cells utilize cysteine biosynthesis through the transsulfuration pathway to support growth (41). The central role of CBS in the transsulfuration pathway and metabolism of sulfur-containing amino acids under physiological conditions is the regulation of CBS in cancer cells, but CBS possesses the capacity to resist the process of ferroptosis that is the consequence of an increase in the level of cellular oxidative stress (42). H_2_S has been implicated in a plethora of physiological and pathological processes. Recent findings indicate that endogenous H_2_S exerts anti-inflammatory and pro-healing effects on intestinal epithelial tissue. Endogenous H_2_S is mainly produced enzymatically by CBS and cystathionine γ-lyase (CSE) in intestinal epithelial cells (37). In IL-10-/-mice, a well-established model of spontaneous colitis, impaired H_2_S synthesis is involved in the exacerbation of colitis associated with hyperhomocysteinemia (43). Accumulating studies have investigated the protective effect of H_2_S on intestinal barrier injuries caused by inflammatory cytokines and lipopolysaccharide in both Caco-2 monolayers (44) and DSS-induced colitis in mice (45, 46).

Our results demonstrate that LPS treatment in Caco-2 cells significantly increases the secretion of pro-inflammatory cytokines (IL-1β, IL-6, TNF-α) and promotes ferroptosis in colonic epithelial cells. TEM revealed characteristic mitochondrial alterations, including swelling and cristae disappearance. Furthermore, hub gene analysis revealed regulatory effects on ferroptosis pathways. Notably, LPS treatment mirrored the effects of the specific ferroptosis inducer RSL3 (a known GPX4 inhibitor that is often used to construct a ferroptotic cell death model) (47), showing elevated iron levels, GSH depletion, and MDA accumulation indicative of lipid peroxidation. Importantly, these effects were reversed by Fer-1 treatment, restoring redox homeostasis and mitochondrial function. These results collectively establish ferroptosis as a key pathological mechanism in LPS-induced enteritis, while highlighting MFN2/CBS signaling as a potential therapeutic target.

Emerging evidence supports the involvement of mitochondrial membrane oxidation in ferroptosis execution (48). Additionally, mitochondria contain 20–50% of total cellular iron, with the mitochondrial iron pool consisting of redox-active iron that exacerbates mitochondrial ROS (mtROS) and lipid peroxidation (LPO) (49). However, the precise mechanisms by which MFN2 modulates ferroptosis remain unclear. Previous studies have shown that MFN2 overexpression inhibited mitochondrial translocation of ACSL4, which ultimately suppressed mitochondria-related iron death (50). Lycopene alleviates multiple-mycotoxin-induced toxicity by inhibiting mitochondrial damage and ferroptosis in the mouse jejunum (51).In contrast, intervention of Chang’an decoction could improve the mucosal barrier integrity and colonic inflammatory response effectively through inhibiting ER stress response mediated by MFN2 (52). Chen et al. demonstrated that MFN2 suppresses ferroptosis by restoring mitochondrial dynamics and bioenergetic homeostasis. Gain-of-function experiments revealed that MFN2 overexpression in CMECs enhanced cell viability, attenuated ROS accumulation, elevated SOD activity and GSH levels, and concurrently decreased iron overload, lipid peroxidation, and LDH release, collectively underscoring the anti-ferroptotic potential of MFN2 (50). Notably, MFN2 likely serves as a critical node linking between mitochondrial integrity, iron metabolism, mitochondrial-ER crosstalk and ferroptosis in IECs.

Liu et al. reported that the activation of the transcription factor 3 (ATF3) has a positive regulatory effect on the CBS in the context of ferroptosis under conditions of cystine deprivation stress. Conversely, the suppression of CBS renders colorectal cancer (CRC) cells more susceptible to ferroptosis by targeting the mitochondrial tricarboxylic acid (TCA) cycle (53). Consequently, the present study hypothesizes that CBS could serve as a viable target to enhance ferroptosis-based therapy. Understanding this biochemical cystine metabolism is essential for studying metabolic functions and potential health implications related to amino acid metabolism.

In addition, the results revealed a significant positive correlation between MFN2 and CBS expression, as well as between different immune cell types, including M1 macrophages, T follicular helper cells and macrophages, in UC. Analysis of immune infiltration patterns revealed an association between elevated expression of the pivotal gene and M1 macrophage activation, given the well-established role of immune cell dysfunction in UC pathogenesis and the importance of ferroptosis in immune cell homeostasis (particularly in T cells and macrophages) (54). The involvement of MFN2 in crosstalk between macrophages and intestinal epithelial cell (IEC) ferroptosis may play a crucial role in the pathogenesis of UC, and this warrants further investigation. Co-culture systems between Caco-2 cells and human mononuclear macrophages could elucidate whether MFN2/CBS knockdown modulates immune cell polarization or cytokine secretion. The molecular mechanisms underlying epithelial-macrophage crosstalk could be clarified by such experiments, which might result in the identification of new therapeutic targets for UC immunotherapy.

Ultimately, bioinformatics analysis and experimental validation revealed that the ferroptosis pathway played a key role in UC response. We also found MFN2 and CBS to be promising candidates for predictive markers, which could be used as a therapeutic target for treating patients with UC.

## Conclusions

In conclusion, this research discovered two genes (MFN2 and CBS) linked with Ferroptosis and could be used as diagnostic markers for UC. Furthermore, these hub genes have been discovered to be interconnected with diverse immune cells, hinting at their potential significance in shaping the immune microenvironment.

## Data Availability

The datasets presented in this study can be found in online repositories. The names of the repository/repositories and accession number(s) can be found in the article/**Supplementary Material**.
