# Supplementary material for: Molecular mechanisms of ferroptosis in ulcerative colitis: insights from machine learning, WGCNA, and immune cell infiltration analysis
Source: Front Immunol. 2025 Aug 29;16:1615186. doi: 10.3389/fimmu.2025.1615186 (PMC12425917; doi:10.3389/fimmu.2025.1615186)
Supplement: Supplementary file 1 [file DataSheet1.zip › Supplementary Materials/Supplementary Materials.pdf]

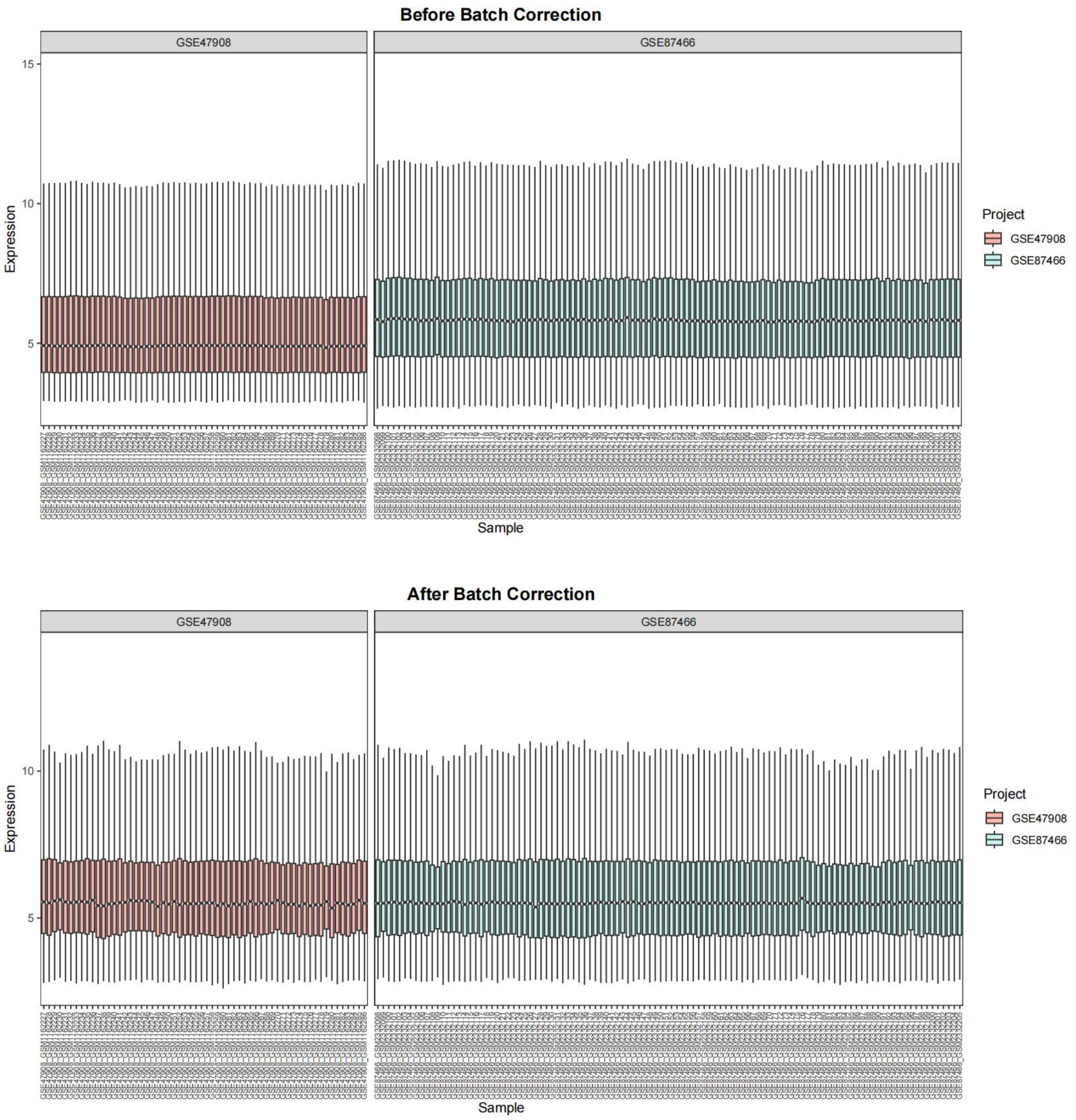

Supplementary Figure 1 The distribution and variations of all gene expression between the UC and control groups are displayed in boxplots (before and after batch correction).

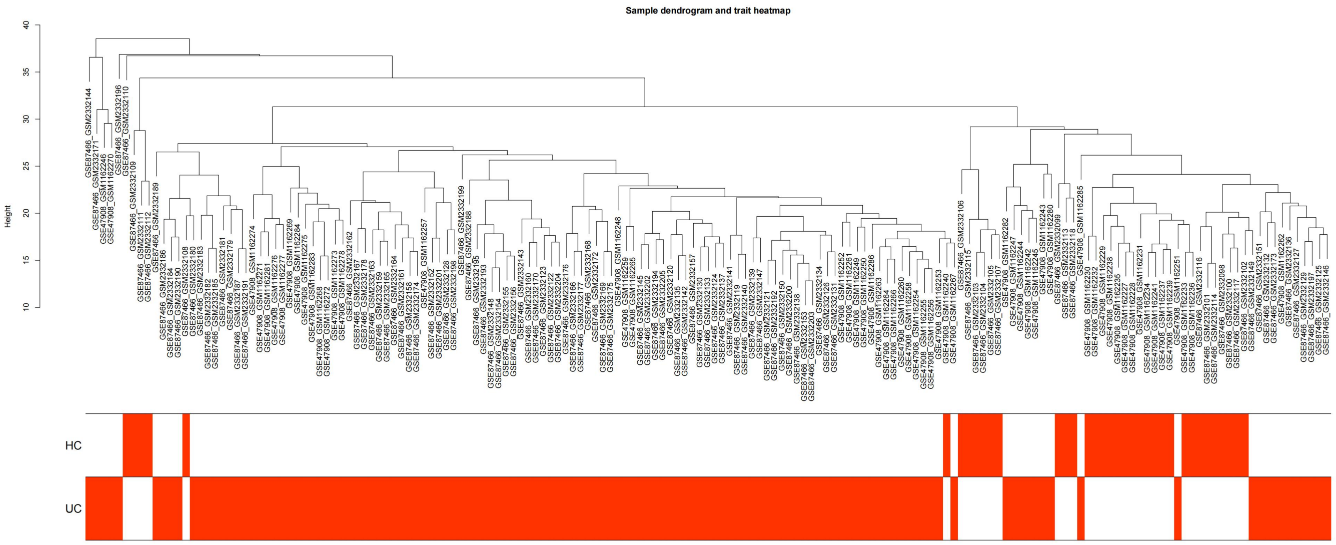

Supplementary Figure 2 Sample clustering and more details results.

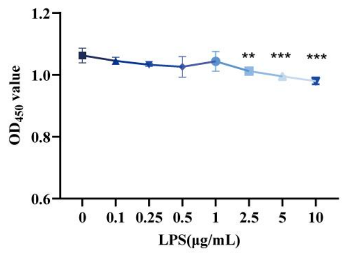

Supplementary Figure 3 Cell viability of Caco-2 cells incubated with LPS (0.1, 0.25, 0.5, 1, 10 μg/mL) for 24 h.
